# *Cinnamomum verum* J. Presl. Bark essential oil: in vitro investigation of anti-cholinesterase, anti-BACE1, and neuroprotective activity

**DOI:** 10.1186/s12906-022-03767-y

**Published:** 2022-11-18

**Authors:** Mina Saeedi, Aida Iraji, Yasaman Vahedi-Mazdabadi, Atiyeh Alizadeh, Najmeh Edraki, Omidreza Firuzi, Mahdieh Eftekhari, Tahmineh Akbarzadeh

**Affiliations:** 1 Medicinal Plants Research Center, Faculty of Pharmacy, Tehran University of Medical Sciences, Tehran, Iran; 2 Persian Medicine and Pharmacy Research Center, Tehran University of Medical Sciences, Tehran, Iran; 3 Stem Cells Technology Research Center, Shiraz University of Medical Sciences, Shiraz, Iran; 4 Central Research Laboratory, Shiraz University of Medical Sciences, Shiraz, Iran; 5 Medicinal and Natural Products Chemistry Research Center, Shiraz University of Medical Sciences, Shiraz, Iran; 6 Department of Pharmacognosy and Pharmaceutical Biotechnology, School of Pharmacy, Kermanshah University of Medical Sciences, Kermanshah, Iran; 7 Department of Medicinal Chemistry, Faculty of Pharmacy, Tehran University of Medical Sciences, Tehran, Iran

**Keywords:** BACE1, Cholinesterase, Essential oil, Inhibitory activity, Neuroprotectivity

## Abstract

**Background:**

*Cinnamomum verum* J. Presl. (Lauraceae), *Myrtus communis* L. (Myrtaceae), *Ruta graveolens* L. (Rutaaceae), *Anethum graveolens* L. (Apiaceae), *Myristica fragrans* Houtt. (Myristicaceae), and *Crocus sativus* L. (Iridaceae) have been recommended for improvement of memory *via* inhalation, in Iranian Traditional Medicine (ITM). In this respect, the essential oils (EOs) from those plants were obtained and evaluated for cholinesterase (ChE) inhibitory activity as ChE inhibitors are the available drugs in the treatment of Alzheimer’s disease (AD).

**Methods:**

EOs obtained from the plants under investigation, were evaluated for their potential to inhibit acetylcholinesterase (AChE) and butyrylcholinesterase (BChE) in vitro based on the modified Ellman’s method. The most potent EO was candidate for the investigation of its beta-secretase 1 (BACE1) inhibitory activity and neuroprotectivity.

**Results:**

Among all EOs, *C. verum* demonstrated the most potent activity toward AChE and BChE with IC_50_ values of 453.7 and 184.7 µg/mL, respectively. It also showed 62.64% and 41.79% inhibition against BACE1 at the concentration of 500 and 100 mg/mL, respectively. However, it depicted no neuroprotective potential against β-amyloid (Aβ)-induced neurotoxicity in PC12 cells. Also, identification of chemical composition of *C. verum* EO was achieved *via* gas chromatography-mass spectrometry (GC-MS) analysis and the major constituent; (*E*)-cinnamaldehyde, was detected as 68.23%.

**Conclusion:**

Potent BChE inhibitory activity of *C. verum* EO can be considered in the development of cinnamon based dietary supplements for the management of patients with advanced AD.

## Background

Alzheimer’s disease (AD) is a chronic neurodegenerative disorder that has been recognized as the key cause of dementia in elderly people. According to the latest data from Centers for Disease Control and Prevention (CDC), 121,499 people died due to AD in 2019. About 1 in 9 people (10.7%) over age 65 suffers from AD dementia and deaths from AD between 2000 and 2019 have more than doubled, increasing by 145%. In 2021, total cost for patients with AD or other types of dementia were was estimated to be $321 billion (https://www.alz.org/media/Documents/alzheimers-facts-and-figures.pdf). The increasing number of patients with AD and the economic burden of the disease have made it one of the greatest challenges of the 21th century.

Currently, there is no definite cure for the treatment of AD due to its multi-factorial nature. Reduced synaptic levels of acetylcholine (ACh) [1], abnormal deposits of β-amyloid peptide (Aβ) [2], intracellular hyper-phosphorylated tau neurofibrillary tangles [3], and mitochondrial dysfunction leading to oxidative stress [4] have been known as the main hallmarks of AD. In this respect, single-target drug therapies have not been successful [5] and recently, the development of multi-target therapeutic agents based on the different mechanisms involved in AD, has absorbed lots of attention [6, 7]. Although using strategies based on the selective ligands have been known as the main tool in the drug discovery developments, it has not been effective in the case of multifactorial diseases such as AD. For this purpose, phytotherapy [8] profiting from valuable biological activities of a mixture of constituents has been in the center of attention. Focusing on the herbal remedies used in the treatment of AD [9], essential oils (EOs) were found to be very effective since various in vitro, in vivo, and clinical trials have endorsed their efficacy because of low molecular weight and high hydrophobicity leading to easy crossing the blood-brain barrier (BBB) [10, 11].

EOs are naturally occurring secondary metabolites which contain a complex mixture of volatile compounds possessing a wide range of biological activities [12, 13].

Various studies have confirmed cholinesterase inhibitory (ChEI) activity [13] and neuroprotectivity of EOs [14, 15]. In addition, the BACE1 inhibitory activity of EOs has been documented in the literature. The enzyme plays a key role in the generation and deposition of neurotoxic β-amyloid peptide (Aβ) (βA). For example, *Lavandula luisieri* EO could efficiently inhibit BACE1 in the enzymatic and cellular assays [16] and *Lavandula angustifolia* EO was also reported to inhibit βA aggregation [17]. The efficacy of *L. angustifolia* and *Rosmarinus officinalis* EOs in the treatment of AD has been clinically investigated and a great improvement in cognitive impairment was reported [17, 18].

It should be noted that aromatherapy using plants such as *Cinnamomum verum* J. Presl. (Lauraceae), *Myrtus communis* L. (Myrtaceae), *Ruta graveolens* L. (Rutaaceae), *Anethum graveolens* L. (Apiaceae), *Myristica fragrans* Houtt. (Myristicaceae), and *Crocus sativus* L. (Iridaceae) has been traditionally recommended for the treatment of memory loss in Iranian traditional medicine (ITM) [19]. On the other hand, non-pharmacological approaches have been developed for the improvement of cognitive problems [20, 21] and in this respect, aromatherapy has been widely considered as a strong tool [22]. In different studies, the combination of therapies has depicted significant results in improving memory, quality of life, and treatment of other complications in patients with AD [23, 24]. Herein, in continuation of our study on the development of herbal remedies for the treatment of AD [25–32], the anti-ChE activity of *Cinnamomum verum* J. Presl., *Myrtus communis* L., *Ruta graveolens* L., *Anethum graveolens* L., *Myristica fragrans* Houtt., and *Crocus sativus* L. EOs were evaluated. In this regard, the most potent EO (*C. verum*) was candidate for the investigation of further biological activities related to AD including neuroprotectivity and BACE1 inhibitory activity.

## Methods

### Plants

Plants including *C. verum* bark (1500 g), the leaves of *M. communis* (1500 g), the aerial parts of *R. graveolens* (1500 g), *A. graveolens* seeds (1500 g), seed kernel of *M. fragrans* (1500 g), and the flowers of *C. sativus* L. (100 g) were bought from the local market in Tehran (Iran), in 2018. They were identified and deposited in the herbarium of Faculty of Pharmacy, Tehran University of Medical Sciences by Professor Gholamreza Amin, with the voucher numbers of pmp-910, pmp-423, pmp-362, pmp-1609, pmp-1608, and pmp-570, respectively. The identification of plants was accomplished by comparison with the identified herbarium and flora specimens.

### Obtaining the essential oils

The EOs of *C. verum* (1000 g), *M. communis* (1000 g), *R. graveolens* (1000 g), *A. graveolens* (1000 g), *M. fragrans* (1000 g), and *C. sativus* (90 g) were obtained using general hydro-distillation in Clevenger-type apparatus. Powdered plant material (250 g) was transferred to 2 L capacity Clevenger apparatus and distilled water was added to the round flask. It should be noted that in the case of *C. sativus*, low amounts of the plant material were used and 90 g of the powdered plant was placed in a 500 mL round flask. Hydro-distillation was conducted for 5–6 h and quitted when the oil stopped condensing. After that the distilled oil was collected, dried over anhydrous sodium sulfate, and then stored in a tightly closed dark vial at 4 °C. The density of all EOs were also calculated.

### Biological activities

#### In vitro AChE and BChE inhibitory activity assay

In vitro anti-ChE activity assays were performed toward acetylcholinesterase (AChE, E.C. 3.1.1.7, Type V-S, lyophilized powder, from electric eel, 1000 unit) and butyrylcholinesterase (BChE, E.C. 3.1.1.8, from equine serum) using the modified Ellman’s method [33]. To obtain acceptable enzyme inhibitory activity (20–80%), the stock solutions of the EOs (10 mg/mL) were prepared in DMSO and were diluted with a mixture of DMSO and methanol to achieve four different final concentrations of the samples (63.5, 125, 250, 500 µg/mL) while obtaining the final ratio of 50/50 DMSO/methanol. Each well contained potassium phosphate buffer (KH_2_PO_4_/ K_2_HPO_4,_ 0.1 M, pH 8) (50 µL), the prepared sample (25 µL) as described above and AChE (25 µL) with the final concentration of 0.22 Units/mL in potassium phosphate buffer (pH = 8). They were pre-incubated for 15 min at room temperature and then of DTNB (5,5′-dithio-bis(2-nitrobenzoic acid) (125 µL, 3 mM in potassium phosphate buffer, pH 8) was added to the mixture. After the addition of substrate (ATCI (acetylthiocholine iodide, 3 mM in distilled water, 25 µL), changes in the absorbance were measured spectrometrically at 405 nm. In parallel, a blank containing all components without enzyme was used to account for the non-enzymatic reaction. A negative control was also performed under the same conditions without inhibitor, and donepezil was used as the positive control. The IC_50_ values were determined graphically from log concentration vs. % of inhibition curves. All experiments were performed in triplicate. The BChE inhibition assay was performed in the same method.

### Neuroprotection assay

Neuroprotective activity of *C. verum* EO on PC12 neuronal cells exposure to Aβ_25−35_ was estimated using the MTT reduction assay according to a previously described method [34]. PC12 (rat pheochromocytoma) cells were a generous gift from Professor Lloyd A. Greene (Department of Pathology and Cell Biology, Columbia University, New York, NY). The PC12 cells were plated on a collagen-coated 96-well plate (5 × 10^5^ cells/mL, 100 µL in each well) and incubated for 48 h at 37 °C. Different concentrations of the test EO were added to each well, incubated for an extra 3 h and then Aβ_25−35_ (5 µM, 10 µL) was added to each well. After 24 h, 90 µL of the medium was replaced with 20 µL of 0.5 mg/mL MTT dissolved in RPMI containing phenol red and incubated for 2 h at 37 °C. Afterward, formazan crystals were solubilized in DMSO (200 µL) and the absorbance (570 nm) was measured using a Bio-Rad microplate reader. Caffeic acid was used as a reference compound.

### In vitro anti-BACE1 activity assay

The preliminary anti-BACE1 activity of the *C. verum* EO was performed by FRET (fluorescence resonance energy transfer) enzyme inhibition. The assay was performed following the previously reported procedures and according to the manufacturer’s instructions (Invitrogen; former Pan Vera corporation, Madison, WI) using OM99-2 as the reference compound [34, 35]. Briefly, stock solutions of *C. verum* EO were prepared in DMSO. Each sample was further diluted in assay buffer to prepare the appropriate concentration of the test sample. The final concentration of DMSO was 6% (v/v). The substrate (10 µL) was added to different concentrations of the test EO (10 µL) in each well of a black 96-well microplate and gently mixed. Then, BACE1 (10 µL) was added to start the reaction. The reaction mixtures were incubated at 25 °C for 90 min in the dark and sodium acetate (2.5 M, 10 µL) was added to stop the reaction. Finally, the fluorescent intensity of the enzymatic product was measured at 544 nm excitation and 590 nm emission wavelengths using BMG, LABTECH, Polar star, Germany.

### Gas chromatography (GC)

Quantification of (*E*)-cinnamaldehyde in the *C. verum* EO was done by external standard method using calibration curves generated by running GC analysis. In this respect, GC analysis of *C. verum* EO and (*E*)-cinnamaldehyde (99% purity) (Sigma-Aldrich, Saint Louis, USA) was carried out on an Agilent 7890 A GC instrument with a flame ionization detector (FID) (Agilent Technologies, CA, USA). The silica capillary column was 19,091 J-433 Agilent (30 × 0.25 mm ID, film thickness: 0.25 μm; CA, USA) and pure nitrogen (99.999%) was applied as the carrier gas at a flow rate of 25 mL/min. The EO sample was injected as 20% *n*-hexane solution (1.0 µL) with the split ratio of 1:60. The initial oven temperature was held for 3 min isothermal at 60 °C and elevated to the maximum of 240 °C at a rate of 6 °C/min for 20 min. Also, *n*-hexane solution of (*E*)-cinnamaldehyde (1.0 µL) was injected with several concentrations of 1000, 2500, 5000, 10,000, 25,000, and 50,000 µg/mL to obtain the calibration curve.

### Gas chromatography-mass spectrometry (GC-MS)

Analysis of *C. verum* EO was achieved on an Agilent 7890 A GC instrument with a flame ionization detector (FID) accompanied with MS-5975 C MSD (Agilent Technologies, CA, USA). The silica capillary column was HP-5ms Agilent (30 × 0.25 mm ID, film thickness: 0.25 𝜇m; CA, USA) and pure helium (99.999%) was applied as the carrier gas at a flow rate of 1 mL/min. Also, injection volume was 1.0 µL with split ratio of 1:5. The initial oven temperature was held 5 min isothermal at 75 °C and increased to 280 °C at a rate of 20 °C min^–1^ for 25 min. Likewise, the MS system was set in electron ionization (EI) mode with a quadrupole detector at 70 eV ionization energy. The transfer line and ion source temperatures were set at 285 and 200 °C, respectively. To identify the chemical composition of EO, the Kovats retention indices of components were calculated using retention times of an *n*-alkane ladder that was injected after EO and mass spectra were compared with spectra available on the computer library.

### Molecular docking study

The gold molecular docking software was employed for docking studies to determine the possible binding site of the seven major constituents of *C. verum* bark EO. The crystal structures of AChE (PDB ID: 4EY7), BChE (PDB ID: 4BDS), BACE1 (PDB ID: 1W51), MAO-A (PDB ID: 2Z5X), and MAO-B (PDB ID: 2XFN) were taken from RCSB-PDB (http://www.rcsb.org). For each code, water molecules and the co-crystallized ligands were removed from the receptor and the co-crystallized inhibitor was retained separately to prepare the protein. Selected compounds for *in silico* assessments were drawn using hyperchem and subjected to energy minimization using MM + and AM1 algorithms. The binding site of the enzymes for the docking process was defined using the native ligands. GOLD docking program with ChemScore function was used for docking analyses. All other options were set as default. The top-score binding poses were used for further analysis. Protein-ligand interactions were analyzed with Discovery Studio Visualizer.

### Statistical analysis

The GraphPad Prism software was used to carry out statistical analysis. Data comparisons were performed by one-way analysis of variance (ANOVA) with Tukey’s multiple comparisons as the post hoc test. *P* values < 0.05 were considered statistically significant.

## Results

The EOs obtained from *C. verum*, *M. communis*, *R. graveolens*, *A. graveolens*, *M. fragrans*, and *C. sativus*, were respectively in dark yellow, light yellow, dark yellow, light cream, colorless, and yellow having density of 0.9766, 0.8592, 0.6568, 0.8996, 0.8422, and 0.9320 g/cm^3^. Also, the extraction yields were calculated as 0.68 (6.8362 g), 0.43 (4.2960 g), 0.06 (0.6568 g), 0.90 (8.9960 g), 0.08 (0.8422 g), and 0.52% (0.4660 g), respectively.

### In vitro anti-ChE activity of EOs

All EOs were evaluated for their AChE and BChE inhibitory activity comparing with donepezil (Table 1), based on the modified Ellman’s method. As can be seen in Table 1, *C. verum* and *M. communis* EOs showed anti-AChE activity with IC_50_ values of 453.7 and 405.1 µg/mL, respectively. Other EOs were inactive toward AChE (IC_50_ > 500 µg/mL). In the case of anti-BChE activity, most EOs were found to be active and *C. verum*, *M. communis*, *R. graveolens*, and *A. graveolen* EOs inhibited BChE with IC_50_ values of 184.7, 431.5, 333.1, and 335.7 µg/mL, respectively. However, *M. fragrans* and *C. sativus* showed no activity. Among EOs, anti-BChE activity of *C. verum* bark EO was significant, hence, it was selected for the evaluation of further biological studies involved in AD. Also, its chemical composition was analyzed *via* GC-MS.

**Table 1:** IC_50_ values (μg/mL) of different plants EOs toward AChE and BChE

Entry	Plants	anti-AChE activity^a^	anti-BChE activity^a,b^
1	*C. verum*	453.7±2.9	184.7±1.1
2	*M. communis*	405.1±3.9	431.5±34.8****
3	*R. graveolens*	> 500	333.1±23.6***
4	*A. graveolen*	> 500	335.7±7.9***
5	*M. fragrans*	> 500	> 500
6	* C. sativus*	> 500	> 500
7	Cinnamaldehyde	> 100^c^	> 100^c^
8	Donepezil	0.020 ± 0.002	1.50 ± 0.27

^a^ IC_50_ values were presented as the mean ± SD of three independent experiments

^b^ Mean comparison of each EO was compared with the value obtained from *C. verum *through one-way ANOVA test followed by Tukey post-hoc multiple comparisons (****p < 0.0001 and ***p < 0.001)

^c^ μM

### Study of neuroprotectivity of *C. verum* bark EO

The neuroprotectivity of *C. verum* bark EO, possessing the best BChE inhibitory activity, was investigated using PC12 cell injury induced by Aβ_25-35_ by MTT assay. It demonstrated no activity compared with caffeic acid as the reference drug.

### In vitro BACE1 inhibitory of *C. verum* bark EO

The *C. verum* bark EO was evaluated for its BACE1 inhibitory activity and compared with OM99-2 as the reference inhibitor. The results are depicted in Table 2.

**Table 2:** BACE1 inhibitory activity of *C. verum* bark EO

Entry	Sample	Inhibition percent	IC_50_ (µM)
1	EO at concentration of 100 mg/mL	62.64%	-
2	EO at concentration of 500 mg/mL	41.79%	-
3	OM99-2^a^	-	0.88 ± 0.66

^a^ Standard BACE1 inhibitor

### Chemical composition of *C. verum* bark EO

GC-MS analysis of *C. verum* bark EO was studied as the most potent BChE inhibitor. The principal compounds, 93.26% of the total essential oil were identified as reported in Table 3. They belonged to different classes of compounds including monoterpenes (0.20%), aldehydes (69.49%), monoterpenoids (0.20%), sesquiterpenes (14.78%), styrenes (4.21%), aromatic carboxylic acids (1.50%), sesquiterpenoids (2.54%), alkane hydrocarbons (0.16%), and aromatic esters (0.18%). Among them, (*E*)-cinnamaldehyde was identified as the most abundant component (68.23%) and δ-cadinene (5.53%), α-copaene (4.25%), cinnamaldehyde dimethyl acetal (4.21%), and α-muurolene (3.8%) were also significant. It should be noted that other components were found to be less than 2%. The presence of dibutyl phthalate seems to be associated with plasticizers contamination [36].

**Table 3:** Chemical composition of *C. verum* bark EO

Entry	Compounds ^1^	RT (min) ^2^	Percent (%) ^3^	RI ^4^	
				Calculated	Literature ^5^
1	α-Pinene^a^	7.56	0.11	950	956
2	Camphene^a^	7.85	0.09	973	981
3	Benzaldehyde^b^	8.08	0.49	992	995
4	3-Phenylpropanal^b^	10.98	0.77	1157	1163
5	Isoborneol^c^	11.05	0.20	1162	1164
6	(*E*)-Cinnamaldehyde^b^	12.41	68.23	1262	1268
7	Cyclosativene^d^	13.15	0.45	1338	1344
8	α-Copaene^d^	13.23	4.25	1343	1349
9	Cinnamaldehyde dimethyl acetal^e^	13.39	4.21	1389	-
10	Isosativene^d^	13.57	0.25	1407	-
11	(*E*)-Cinnamic acid^f^	13.74	1.50	1420	1428
12	Aromadendrene^d^	13.82	0.26	1431	1439
13	(*Z*)-α-Bisabolene^d^	13.94	0.23	1469	1476
14	α-Muurolene^d^	14.30	3.18	1471	1479
15	δ-Cadinene^d^	14.49	5.53	1497	1503
16	β-Oplopenone^g^	14.88	0.28	1580	1585
17	δ-Cadinol^g^	15.21	0.64	1637	1644
18	t-Cadinol^g^	15.46	1.26	1645	1649
19	t-Muurolol^g^	15.57	0.36	1650	1650
20	Cadalene^d^	15.72	0.63	1662	1665
21	Octadecane^h^	16.42	0.16	1796	1800
22	Dibutyl phthalate^i,6^	16.99	0.18	1897	1906
	Total (%) identified		93.26		

^1^ The chemical composition was identified based on the MS spectra and Kovats retention indices. Compounds belong to different classes including monoterpenes ^a^, aldehydes ^b^, monoterpenoids ^c^, sesquiterpenes ^d^, styrenes ^e^, carboxylic acids ^f^, sesquiterpenoids ^g^, alkane hydrocarbons ^h^, and aromatic esters ^i^

^2^ Retention time

^3^ Detected based on the GC (FID)

^4^ Kovats retention index

^5^ All calculated RI values were compared with those reported in the literature at the same conditions, cited in PubChem and NIST (National Institute of Standards and Technology)

^6^It may be associated with the presence of plasticizers

### The quantity of (*E*)-cinnamaldehyde in *C. verum* bark EO

According to the GC-MS analysis, (*E*)-cinnamaldehyde was the most abundant constituent. The results demonstrated correlation coefficients (r^2^) greater than 0.996 in the range of 1000–50,000 mg/mL. The (*E*)-cinnamaldehyde peak was detected at 13.10 min and the quantity was calculated as 1.1⋅10^5^ µg/mL using calibration curve as explained in the experimental section.

### Molecular docking study

The most abundant compounds reported in *C. verum* bark EO (Table 3) including δ-cadinene, t-cadinol, (*E*)-cinnamaldehyde, cinnamaldehyde dimethyl acetal, (*E*)-cinnamic acid, α-copaene, and α-muurolene were considered by an *in silico* approach to be investigated against AChE, BChE, BACE1, MAO-A, and MAO-B which are implicated in the pathogenesis of AD (Tables 4, 5, 6, 7 and 8).

**Table 4:** Docking study of the most abundant compounds in the *C. verum* bark EO against AChE

Compound	Gold score	Residues	Interactions
δ-Cadinene	60.89	Leu289	Alkyl
		Trp286	Alkyl
		Trp286	Alkyl
		Trp286	Pi-Alkyl
		Phe338	Alkyl
		Tyr341	Pi-Sigma
		Tyr341	Alkyl
t-Cadinol	66.84	Tyr72	Alkyl
		Tyr72	Pi-Alkyl
		Tyr124	Pi-Alkyl
		Tyr341	H-Bonding
		Tyr341	Alkyl
		Tyr341	Alkyl
		Tyr341	Pi-Alkyl
		Trp286	Pi-Alkyl
		Trp286	Alkyl
		Phe338	Alkyl
		Phe338	Alkyl
		Val294	Alkyl
(*E*)-Cinnamaldehyde	46.97	Tyr72	H-Bonding
		Phe297	Pi-Pi Stacked
		Trp286	Pi-Pi Stacked
		Tyr341	Pi-Pi Stacked
Cinnamaldehyde dimethyl acetal	56.76	Phe297	Pi-Pi Stacked
		Phe338	H-Bonding
		Tyr341	Pi-Pi Stacked
*(E)*-Cinnamic acid	52.02	Trp86	Pi-Pi Stacked
		Asp74	H-Bonding
		Tyr337	H-Bonding
		Tyr341	H-Bonding
		Tyr341	H-Bonding
α-Copaene	54.85	Trp86	Pi-Alkyl
		Trp86	Pi-Alkyl
		Trp86	Pi-Alkyl
		Trp86	Pi-Alkyl
		Tyr337	Pi-Alkyl
		Tyr337	Pi-Alkyl
		Tyr337	Pi-Alkyl
		Phe338	Pi-Alkyl
		Phe338	Pi-Alkyl
		Tyr341	Pi-Alkyl
		Tyr341	Pi-Alkyl
		His447	Pi-Alkyl
		His447	Pi-Alkyl
α-Muurolene	63.23	Trp86	Pi-Sigma
		Trp86	Pi-Alkyl
		Trp86	Pi-Alkyl
		Tyr337	Pi-Sigma
		Tyr337	Pi-Alkyl
		Tyr341	Pi-Alkyl
		Tyr341	Pi-Alkyl
		Phe338	Pi-Alkyl
		His447	Pi-Alkyl
		His447	Pi-Alkyl

**Table 5:** Docking study of the most abundant compounds in the *C. verum* bark EO against BChE

Compound	Gold score	Residues	Interactions
δ-Cadinene	49.89	Trp82	Pi-Alkyl
		Trp82	Pi-Alkyl
		Trp82	Pi-Alkyl
		Trp82	Pi-Alkyl
		Tyr128	Pi-Alkyl
		His438	Pi-Alkyl
t-Cadinol	53.46	Trp82	Pi-Alkyl
		Trp82	Pi-Sigma
		Ala328	Alkyl
		His438	Alkyl
(*E*)-Cinnamaldehyde	44.42	Trp82	Pi-Pi Stacked
		His438	Pi-Pi Stacked
Cinnamaldehyde dimethyl acetal	48.17	Trp82	Pi-Pi Stacked
		Trp332	H-Bonding
*(E)*-Cinnamic acid	54.52	Asp70	H-Bonding
		Trp82 Trp332 His438	Pi-Pi Stacked H-Bonding Pi-Pi Stacked
α-Copaene	49.48	Trp82	Pi-Alkyl
		Trp82	Pi-Alkyl
		Trp82	Pi-Alkyl
		Trp82	Alkyl
		Trp82	Alkyl
		Trp430	Pi-Alkyl
		Ala328	Pi-Alkyl
		Ala328	Alkyl
		Phe329	Alkyl
		Tyr332	Alkyl
α-Muurolene	48.53	Trp82	Pi-Alkyl
		Trp82	Alkyl
		Trp82	Pi-Sigma
		Tyr440	Alkyl
		Ala328	Alkyl
		Ala328	Pi-Alkyl
		Phe329	Alkyl
		His438	Pi-Alkyl
		His438	Alkyl

**Table 6:** Docking study of the most abundant compounds in the *C. verum* bark EO against BACE1

Compound	Gold score	Residues	Interactions
δ-Cadinene	47.55	Tyr71	Pi-Alkyl
		Tyr71	Pi-Alkyl
		Phe108	Pi-Alkyl
		Trp115	Pi-Alkyl
t-Cadinol	42.14	Tyr71	Pi-Alkyl
		Tyr71	Pi-Alkyl
		Gln73	H-Bonding
(*E*)-Cinnamaldehyde	38.09	Tyr71	H-Bonding
		Ile118	Pi-Alkyl
Cinnamaldehyde dimethyl acetal	43.77	Trp71 Asp228	Pi-Pi Stacked H-Bonding
*(E)*-Cinnamic acid	46.22	Thr72	H-Bonding
		Tyr71	Pi-Pi Stacked
		Ile126	Pi-Alkyl
α-Copaene	44.32	Leu30	Alkyl
		Tyr71	Alkyl
		Tyr71	Alkyl
		Tyr71	Alkyl
		Phe108	Pi-Alkyl
		Phe108	Pi-Alkyl
		Phe108	Pi-Alkyl
		Trp115	Alkyl
α-Muurolene	42.45	Trp71	Pi-Alkyl
		Trp71	Alkyl
		Trp71	Alkyl
		Trp71	Alkyl
		Trp198	Alkyl
		Ile226	Alkyl

**Table 7:** Docking study of the most abundant compounds in the *C. verum* bark EO against MAO-A

Compound	Gold score	Residues	Interactions
δ-Cadinene	47.31	Ile180	Alkyl
		Ile180	Alkyl
		Ile180	Pi-Alkyl
		Phe208	Alkyl
		Phe208	Alkyl
		Cys323	Alkyl
		Cys323	Pi-Alkyl
		Ile335	Pi-Alkyl
		Ile335	Alkyl
		Leu337	Pi-Alkyl
		Leu337	Pi-Alkyl
		Leu337	Alkyl
t-Cadinol	46.96	Tyr69	Alkyl
		Ile207	H-Bonding
		Ile335	Alkyl
		Ile335	Pi-Alkyl
		Leu337	Alkyl
		Leu337	Pi-Alkyl
		Met350	Alkyl
		Tyr407	Alkyl
		Tyr407	Alkyl
		Tyr444	Alkyl
		Phe352	Alkyl
		Gln215	Unfavourable
(*E*)-Cinnamaldehyde	36.37	Tyr407	Van der Waals
Cinnamaldehyde dimethyl acetal	52.21	Tyr69	H-Bonding
		Phe208	Pi-Pi T-Shaped
		Ile335	Pi-Sigma
		Leu337 Tyr407 Phe352	Pi-alkyl H-Bonding H-Bonding
*(E)*-Cinnamic acid	46.43	Asn181	H-Bonding
		Tyr444	H-Bonding
		Tyr444	H-Bonding
		Leu337	Pi-Alkyl
		Ile335	Pi-Alkyl
		Met350	Pi-Sulfur
α-Copaene	43.60	Tyr407	Alkyl
		Tyr407	Alkyl
		Tyr444	Alkyl
		Tyr69	Alkyl
		Phe352	Alkyl
		Phe352	Alkyl
		Leu337	Alkyl
		Ile335	Alkyl
		Ile335	Pi-Alkyl
		Ile335	Alkyl
		Ile335	Pi-Alkyl
		Ile180	Alkyl
		Ile180	Pi-Alkyl
		Phe208	Alkyl
α-Muurolene	45.46	Tyr407	Alkyl
		Ile180	Alkyl
		Ile180	Pi-Alkyl
		Phe208	Alkyl
		Phe208	Pi-Alkyl
		Ile325	Alkyl
		Ile335	Alkyl
		Ile335	Pi-Alkyl
		Val210	Alkyl
		Phe352	Pi-Alkyl
		Ile337	Pi-Alkyl
		Ile337	Alkyl
		Met350	Unfavourable
		Met350	Unfavourable

**Table 8:** Docking study of the most abundant compounds in the *C. verum* bark EO against MAO-B

Compound	Gold score	Residues	Interactions
δ-Cadinene	50.37	Leu164	Alkyl
		Leu164	Alkyl
		Trp119	Alkyl
		Leu167	Alkyl
		Ile316	Alkyl
		Ile316	Pi-Alkyl
		Phe168	Alkyl
		Phe168	Alkyl
		Cys172	Pi-Alkyl
		Leu171	Pi-Alkyl
		Leu171	Alkyl
		Tyr326	Pi-Alkyl
		Tyr326	Alkyl
		Ile199	Alkyl
		Ile199	Alkyl
		Ile199	Pi-Alkyl
t-Cadinol	46.64	Leu88	Alkyl
		Pro104	Alkyl
		Pro104	Alkyl
		Phe103	Alkyl
		Phe103	Pi-Alkyl
		Leu164	Alkyl
		Leu164	Pi-Alkyl
		Ile199	Pi-Alkyl
		Trp199	Alkyl
		Trp199	Pi-Alkyl
		Ile316	Pi-Alkyl
		Tyr326	Pi-Alkyl
(*E*)-Cinnamaldehyde	46.25	Leu164	Pi-Alkyl
		Ile199	H-Bonding
		Ile316	Pi-alkyl`
		Tyr326	H-Bonding
Cinnamaldehyde dimethyl acetal	52.57	Ieu171	Pi-Sigma
		Cys172	Pi-Sulfur
		Ile199	Pi-alkyl`
		Tyr326	Pi-Pi T-shaped
*(E)*-Cinnamic acid	51.41	Pro102	H-Bonding
		Leu164	Pi-Alkyl
		Leu167	Pi-Alkyl
		Phe168	Pi-Pi T-shaped
		Ile199	Pi-Alkyl
		Ile316	Pi-Alkyl
		Tyr326	H-Bonding
		Trp119	Pi-Alkyl
α-Copaene	50.05	Phe168	Pi-Alkyl
		Phe168	Alkyl
		Cys172	Alkyl
		Leu171	Pi-Alkyl
		Leu171	Alkyl
		Leu171	Alkyl
		Leu167	Alkyl
		Leu167	Alkyl
		Leu164	Pi-Alkyl
		Ile199	Alkyl
		Ile199	Alkyl
		Ile199	Pi-Alkyl
		Ile316	Alkyl
		Ile316	Alkyl
		Ile316	Pi-Alkyl
		Ile316	Pi-Alkyl
α-Muurolene	51.32	Leu167	Alkyl
		Leu164	Alkyl
		Phe168	Alkyl
		Leu171	Alkyl
		Leu171	Pi-Alkyl
		Cys172	Alkyl
		Ile199	Alkyl
		Ile199	Alkyl
		Ile199	Pi-Alkyl
		Ile199	Pi-Alkyl
		Ile316	Alkyl
		Ile316	Alkyl
		Ile316	Pi-Alkyl
		Tyr326	Alkyl

According to Tables 4 and 5 which are related to the inhibition of AChE and BChE by the selected compounds, those constituents effectively participated in the interaction with those enzymes with gold score values of 66.84 to 46.97 and 54.52 to 44.42, respectively. Also, they exhibited gold score values in the range of 47.55 to 38.09 against BACE1 (Table 6). In this series of compounds, δ-cadinene and (*E*)-cinnamic acid recorded the best affinity with gold score values of 47.55 and 46.22, respectively. In the case of MAO-A (Table 7), the gold score values were recorded in the range of 52.21 to 36.37. In this group, cinnamaldehyde dimethyl acetal showed the best affinity to the enzyme. Finally, the most abundant compounds in *C. verum* bark EO demonstrated gold score values of 52.57 to 46.25 against MAO-B (Table 8). In this respect, cinnamaldehyde dimethyl acetal (gold score = 52.57), *(E)*-cinnamic acid (gold score = 51.41), and α-muurolene (gold score = 51.32) were found to be the most potent compounds in this series.

## Discussion

AD is a progressive neurodegenerative disorder disease that needs multiple therapeutic approaches. In recent years, aromatherapy has been found as an efficient tool to reduce cognitive impairment resulting from the disease AD [22–24]. For this purpose, we focused on the activity of *C. verum* bark, the leaves of *M. communis*, the aerial parts of *R. graveolens*, *A. graveolens* seeds, seed kernel of *M. fragrans*, and the flowers of *C. sativus* EOs as they were recommended to improve memory *via* aromatherapy in ITM [19]. It is worth mentioning that the anti-AD activity of *Cinnamomum* sp. has been proven through various mechanisms in the clinical or preclinical studies indicating that cinnamon is essential for developing drugs for neurodegenerative disorders [37].

EOs have shown a promising ChE inhibitory activity and various compounds such as α-pinene are responsible for inducing desired activity [13]. According to the literature, the methanol extract of *Cinnamomum zeylanicum* leaves (from local market in Kolkata-India) showed good activity toward AChEI and BChEI with IC_50_ values of 77.78 and 88.62 µg/mL, respectively compared with galantamin (IC_50_s = 22.34 and 25.35, respectively) [38]. Also, the corresponding values for the isolated oil were reported as IC_50_s = 45.88 and 87.39 µg/mL, respectively. It seems that the methanolic extract was found to be more potent than the essential oil. Tepe et al. recorded the AChE and BChE inhibitory activity of *C. zeylanicum* barks EO (from local market in Kilis-Turkey) as 99.68 and 99.18% at the concentration of 20 mg/mL, compared with galantamine with inhibition percent of 89.4 and 74.83%, respectively [39]. Considering the fact that the positive controls in these reports and our study are different, more discussion can’t be provided for the comparison of EOs potency. It is apparent that the EOs composition percent depends on the geographical area and climatic conditions of the plant collection site. In this study, good and selective BChEI activity of *C. verum* EO would be important in the treatment of patients with late-stage AD as the presence of this enzyme at high concentration in severe/late stages of AD has been indicated [40].

As (*E*)-cinnamaldehyde is the most abundant component of *C. verum* EO, it was also evaluated for its anti-ChE activity (Table 1, Entry 7), whether it is responsible for the corresponding inhibitory activity or not. It displayed no inhibitory activity toward both AChE and BChE, consequently, the activity of *C. verum* EO is probably related to the synergistic effects of several compounds.

Cinnamon has shown an important protection against AD and dementia. For example, administration of the hydroalcoholic extract of *C. verum* bark at the concentration of 200 mg/kg for 21 days showed significantly impaired acquisition and retention of memory in the scopolamine-induced memory impairment in experimental rat model [41]. Also, the cinnamon polyphenol extract at the concentration of 10 mg/kg effectively reduced infarct and edema formation in traumatic brain injury in male mice. The remarkable role of brain trauma has been identified as a significant risk factor for the development of AD [42]. However, there is no report on the neuroprotectivity of cinnamon oil. In this study, *C. verum* bark EO exerted no neuroprotective activity against β-amyloid (Aβ)-induced neurotoxicity in PC12 cells. Although the role of (*E*)-cinnamaldehyde in a MPTP (1-methyl-4-phenyl-1,2,3,6-tetrahydropyridine) mouse model of Parkinson’s disease [43] and glutamate-induced oxidative stress on PC12 cells [44] has been documented in the literature, it apparently played no role in the protection of PC12 cell injury induced by Aβ_25−35_.

BACE1 inhibitory activity of *C. verum* has not been investigated in the literature and herein we found that it indicated the percentage inhibition of 62.64% and 41.79% at 500 and 100 mg/mL, respectively, compared with OM99-2 (IC_50_ = 0.88 ± 0.66 µM). However, there is a report by Kang et al. [45] which indicated that the methanol extract of cinnamon bark at the concentration of 100 µg/mL reduced the production of Aβ40 in Chinese hamster ovarian (CHO) cells stably expressing amyloid precursor protein (APP) as determined by enzyme-linked immunosorbent assay. β-Amyloid (Aβ) is produced *via* the amyloidogenic pathway from APP by β-secretase and γ-secretase. Among six phenylpropanoids were isolated from the extract, cryptamygin A reduced Aβ40 production by 60%, at the concentration of 4 µg/mL.

Finally, molecular docking study was performed to get better insight into the interaction of the EO components and different targets involved in AD. In this respect, the interaction of δ-cadinene, t-cadinol, (*E*)-cinnamaldehyde, cinnamaldehyde dimethyl acetal, (*E*)-cinnamic acid, α-copaene, and α-muurolene with various enzymes including AChE, BChE, BACE1, MAO-A, and MAO-B which are responsible in the creation and progression of AD, was studied. Focusing on the gold score values (Tables 4, 5, 6, 7 and 8), desired interactions between these compounds and AChE as well as BChE were constructed. Also, δ-cadinene and (*E*)-cinnamic acid showed the best affinity to BACE1 which endorses in vitro results in our study. Cinnamaldehyde dimethyl acetal was found to be a potent inhibitor of MAO-A and MAO-B, however, (*E*)-cinnamic acid and α-muurolene efficiently could inhibit MAO-B. It depicted that cinnamon oil could be a potent inhibitor of MAO-A and MAO-B. It has been reported that the *C. zeylanicum* EO possessed MAO-A and MAO-B inhibitory activity with inhibition percent of 96.44 and 96.32%, respectively, at a concentration of 2.0 mg/mL, compared with rasagiline (97.42 and 97.8%, respectively) [39].

## Conclusion

In conclusion, the essential oil of *C. verum* can be considered in the management of AD symptoms as good ChE and BACE1 inhibitory activity, were obtained. It may be useful for the aromatherapy of patients with late-stage AD as better BChEI (IC_50_ = 184.7 µg/mL) activity was reported comparing with AChEI activity (IC_50_ = 453.7 µg/mL).

## Data Availability

All relevant data are included within the manuscript and are available from the corresponding author on reasonable request.

## Abbreviations

AChE: Acetylcholinesterase
AD: Alzheimer’s disease
Aβ: β-Amyloid
BACE1: beta-Secretase 1
BChE: Butyrylcholinesterase
ChE: Cholinesterase
EO: Essential oil
GC-MS: Gas chromatography-mass spectroscopy
ITM: Iranian Traditional Medicine
MAO-A: Monoamine oxidase A
MAO-B: Monoamine oxidase B
