# Correction: *Cinnamomum verum* J. Presl. Bark essential oil: in vitro investigation of anti-cholinesterase, anti-BACE1, and neuroprotective activity

**DOI:** 10.1186/s12906-022-03809-5

**Published:** 2022-11-29

**Authors:** Mina Saeedi, Aida Iraji, Yasaman Vahedi-Mazdabadi, Atiyeh Alizadeh, Najmeh Edraki, Omidreza Firuzi, Mahdieh Eftekhari, Tahmineh Akbarzadeh

**Affiliations:** 1 Medicinal Plants Research Center, Faculty of Pharmacy, Tehran University of Medical Sciences, Tehran, Iran; 2 Persian Medicine and Pharmacy Research Center, Tehran University of Medical Sciences, Tehran, Iran; 3 Stem Cells Technology Research Center, Shiraz University of Medical Sciences, Shiraz, Iran; 4 Central Research Laboratory, Shiraz University of Medical Sciences, Shiraz, Iran; 5 Medicinal and Natural Products Chemistry Research Center, Shiraz University of Medical Sciences, Shiraz, Iran; 6 Department of Pharmacognosy and Pharmaceutical Biotechnology, School of Pharmacy, Kermanshah University of Medical Sciences, Kermanshah, Iran; 7 Department of Medicinal Chemistry, Faculty of Pharmacy, Tehran University of Medical Sciences, Tehran, Iran

**Correction: BMC Complement Med Ther 22, 303 (2022)**

**https://doi.org/10.1186/s12906-022-03767-y**

Following publication of the original article [1], the authors reported that some corrections which were indicated in the proof have not been corrected in the published article.

The updated text are given below and the changes have been highlighted in **bold typeface**.

**Background**

In 2021, total cost for patients with AD or other types of dementia **was** estimated to be $321 billion (https://www.alz.org/media/Documents/alzheimers-factsand-figures.pdf).

**Discussion**

AD is a progressive neurodegenerative **disorder** that needs multiple therapeutic approaches. In recent years, aromatherapy has been found as an efficient tool to reduce cognitive impairment resulting from the **disease** [22–24].

The original article [1] has been updated.
